# Acknowledgement to Reviewers of *Diseases* in 2019

**DOI:** 10.3390/diseases8010003

**Published:** 2020-01-19

**Authors:** Diseases Editorial Office

**Affiliations:** MDPI, St. Alban-Anlage 66, 4052 Basel, Switzerland

The editorial team greatly appreciates the reviewers who have dedicated their considerable time and expertise to the journal’s rigorous editorial process over the past 12 months, regardless of whether the papers are finally published or not. In 2019, a total of 51 papers were published in the journal, with a median time to first decision of 22 days and a median time from submission to publication of 47 days. The editors would like to express their sincere gratitude to the following reviewers for their generous contribution in 2019:
Abraham, Bincy P.Karim, ShahidAlao, John PatrickKojima, ShotaAl-Hasan, Majdi N.Koppe, LaetitiaAnastasi, EmanuelaKrishnakumar, DevadasArndt, Patrick G.Kujawska, MałgorzataAssari, ShervinKumar, AnandAzarnia Tehran, DomenicoLajolo, CarloBakillah, Ahmedlastname, firstnameBanerjee, KalpitaLavdaniti, MariaBanning, AntjeLavender, AndrewBattino, MaurizioLavie, Carl J.Bentel, Jacqueline M.Lee, Huei-JaneBiderman, MichaelLegartová, SoňaBillingsley, Hayley E.Lévêque, NicolasBlackall, PatrickLinares, José Jesús GázquezBorecki, MichalLuce-Fedrow, AlisonBottoms, LindsayMadeira De Carvalho, Luis ManuelBrusini, PaoloManciulli, TommasoBuch, ShilpaMarks, Robert S.Buigues, CristinaMascitti, MarcoCalistri, AriannaMaurya, ShailendraCampbell, DanielMediouni, SoniaCarneiro, Sueli Coelho Da SilvaMetz, Stefan W.Cauli, OmarMiletta, MariaCausin, PaolaMitra, Amal K.Ceranowicz, PiotrMlocicki, DanielCerejeira, JoaquimMonroy, FernandoChaaban, HalaMonsuez, Jean-JacquesChang, Chuang-RungMorel, BenoitChen, GuoxunMukaetova-Ladinska, Elizabeta BChen, Kow-TongNielsen, Ole HaagenChen, TaipingNifli, Artemissia-PhoebeChen, XiNissapatorn, VeeranootChien, Yin-HsiuNoonan, VikkiChou, C. JamesOlova, NellyChover, ElenaOon, Hazel H.Claus, ClaudiaOvejero-Benito, María CarmenConte, MariarosariaPagé, GabrielleCorreia Coelho, Ana CláudiaPettigrew, MarkCorreia Santos, NadinePetzke, MaryCuevas-Covarrubias, SergioPongratz, GeorgCullen, PaulPoulton, Alison SallyDe Berardis, DomenicoRayner, BenDi Giallonardo, FrancescaRego, RyanDrosos, A. A.Reipa, VytasEboigbodin, KevinRobinson, EdwardEdlefsen, Paul T.Ronsard, LaranceEilertsen, Karl-ErikRoques, ChristineFeinn, Richard S.Rosenthal, KenFischer, KatjaSaboga Nunes, LuisFlorescu, MonicaSapone, AnnaFrade, João Manuel GraçaSato, Marcello OtakeFrentiu, FrancescaScioscia, CrescenzioFrisher, MartinSiniscalco, DarioFrolov, AndrejSmith, Tara C.Gaitskell, KeziaSoares, MarceloGetahun, AndrewTakuma, KazuhiroGłąbska, DominikaTeschke, RolfGoffart, SteffiThomas-Hawkins, CharlotteGomez-Pinilla, Pedro J.Thornley, SimonGoto, KatsumasaTiwari, VaibhavGuerini, Franca R.Tonetti, LorenzoGuo, WeiminTryndyak, VolodymyrHeggermont, WardVajro, PietroHenry, Ryan A.Vasin, Mikhail V.Hinojosa, Melanie SbernaVenkatesh, SiddarthHo CM, RogerVeraldi, StefanoHo, MengfeiVitetta, LuisHo, Roger C.Vlachou, EugeniaHoff, RodneyWall Medrano, AbrahamHsu, LewisWang, ZenengHuang, Guan-JhongWard, DouglasIsozaki, TakeoWebb, Cameron E.Jee, DonghyunYamaguchi, SatoshiJeverica, SamoYamauchi, YoheiJi, YuelongYang, XiaoheKamimura, HiroteruYero, DanielKanda, TatsuoZuhl, MicahKaranis, Panagiotis


